# Detecting RNA-RNA interactions in *E. coli* using a modified CLASH method

**DOI:** 10.1186/s12864-017-3725-3

**Published:** 2017-05-03

**Authors:** Tao Liu, Kaiyu Zhang, Song Xu, Zheng Wang, Hanjiang Fu, Baolei Tian, Xiaofei Zheng, Wuju Li

**Affiliations:** 1 Beijing Institute of Basic Medical Sciences, Taiping Road 27, Haidian district Beijing, 100850 China; 2 Beijing Institute of Radiation Medicine, Taiping Road 27, Haidian district Beijing, 100850 China

**Keywords:** RNA-RNA interaction, High-throughput sequencing, Bacteria

## Abstract

**Background:**

Bacterial small regulatory RNAs (sRNAs) play important roles in sensing environment changes through sRNA-target mRNA interactions. However, the current strategy for detecting sRNA-mRNA interactions usually combines bioinformatics prediction and experimental verification, which is hampered by low prediction accuracy and low-throughput. Additionally, among the 4736 sequenced bacterial genomes, only about 2164 sRNAs from 319 strains have been described. Furthermore, target mRNAs of only 157 sRNAs have been uncovered. Obviously, highly efficient methods were required to detect sRNA-mRNA interactions in the sequenced genomes. This study aimed to apply a modified CLASH (cross-linking, ligation and sequencing hybrids) method to detect RNA-RNA interactions in *E. coli*, a model bacterial organism.

**Results:**

Statistically significant interactions were detected in 29 transcript pairs. To the best of our knowledge, 24 pairs were reported for the first time and were novel RNA interactions, including tRNA-tRNA, tRNA-ncRNA (non-coding RNA), tRNA-rRNA, rRNA-mRNA, rRNA-ncRNA, rRNA-rRNA, rRNA-IGT (intergenic transcript), and tRNA-IGT interactions.

**Conclusions:**

Discovery of novel RNA-RNA interactions in the present study demonstrates that RNA-RNA interactions might be far more complicated than ever expected. New methods may be required to help discover more novel RNA-RNA interactions. The present work describes a high-throughput protocol not only for discovering new RNA interactions, but also directly obtaining base-pairing sequences, which should be useful in assessing RNA structure and interactions.

**Electronic supplementary material:**

The online version of this article (doi:10.1186/s12864-017-3725-3) contains supplementary material, which is available to authorized users.

## Background

RNA-RNA interactions (RRIs) play important roles in multiple biological processes. For example, mounting evidence suggests that miRNA-mRNA interactions in eukaryotes and sRNA-mRNA interactions in bacteria can exert posttranscriptional regulation of gene expression [1–3]. In addition, snoRNAs can guide chemical modifications of rRNAs through snoRNA-rRNA interactions [2]. Furthermore, many other types of RRIs have been found, and include miRNA-lncRNA, lncRNA-mRNA, lncRNA-lncRNA, snoRNA-mRNA, and scaRNA-rRNA interactions [4–7]. Therefore, detecting RRIs, especially transcriptome-wide RRIs, is an important strategy to understand RNA functions and related biological processes. To this end, many bioinformatics and experimental approaches have been developed.

The current bioinformatics methods for predicting RRIs take RNA sequences as input, and can be divided into two classes [8]. The first class comprises general models for the prediction of RRIs. For example, the earliest methods, e.g. RNAfold [4] or Mfold [5], detect RRIs by predicting the secondary structure of a combined RNA sequence, which is composed of two RNAs to be studied. Base-pairing regions between the two RNAs can demonstrate their interaction. Meanwhile, complex models such as RNAcofold [4] and RNAplex [6] have been developed. These models can be applied to detect binding sites between two RNA molecules, but cannot be applied to determine whether two RNAs interact directly or not. The second class is specially designed for particular RNA types such as miRNAs or bacterial sRNAs. For instance, multiple models have been developed for miRNA target prediction [7, 9, 10]: TargetScan [11], PicTar [12], PITA [13], rna22 [14], and RNAhybrid [15]. In addition, there are programs designed for sRNA-target mRNA prediction, including IntaRNA [16], CopraRNA [16], RNApredator [17], TargetRNA2 [18], sRNATarget [19], and sTarPicker [20]. These models often provide candidate interactions for experimental validation. However, the main shortcoming of such models is the high false positive rate [7, 8]. To overcome this, high-throughput sequencing (HITS)-based protocols have been developed to detect RRIs.

An early strategy is the high-throughput sequencing of RNAs isolated by crosslinking immunoprecipitation(HITS-CLIP), which was developed to decode miRNA-mRNA interactions in the mouse brain [21]. At first, two HITS datasets, Ago-miRNA and Ago-mRNA, were generated respectively. Then, bioinformatics methods were developed to predict miRNA-mRNA interactions. Photoactivatable-ribonucleoside-enhanced crosslinking and immunoprecipitation (PAR-CLIP) was next developed [22]; in this method, 4-thiouridine is used to introduce thymidine to cytidine transitions during cDNAs library preparation. The information of transitions could be used to determine miRNA target sites. However, the recently-developed CLASH (cross-linking, ligation and sequencing hybrids) or iPAR-CLIP (in vivo PAR-CLIP) method is more effective in directly detecting RRIs. The key idea behind the CLASH or iPAR-CLIP is to identify chimeric reads formed by RRIs. So far, the CLASH method has been applied to identify snoRNA-rRNA interactions in yeast [23], sRNA-RNA interactions in bacteria [24, 25], and miRNA-mRNA interactions in humans [26]. Additionally, the iPAR-CLIP method was applied to assess miRNA-target interactions in *C. elegans* [27]. However, these methods used specific proteins to detect RRIs only associated with them, which did not cover the whole RNA-RNA interactome. This study aimed to apply a modified CLASH strategy to assess all RRIs in *E. coli*, it doesn’t matter if they are associated with other partner molecules such as proteins. To the best of our knowledge, CLASH or similar methods have not been applied to detect transcriptome-wide whole RRIs in prokaryotes.

## Methods

### UV cross-linking of living cells treated with AMT

*Escherichia coli K12 MG1655* cells were centrifuged and washed with PBS, resuspended with PBS at a density of 5 × 10^9^ cells/ml and incubated on ice. AMT (Sigma) was added to treat the cells at a concentration of 0.3 mg/ml, on ice for 10 min. Then, the cells were kept on ice and subjected to UV irradiation at 365 nm with an intensity of 10 mW/cm^2^ six times (10 min each); cells were shaken well before irradiation.

### Cell lysis and RNA extraction

After cross-linking, cells were washed twice with PBS. Lysozyme solution (TIANGEN) and 10% SDS (Sigma) were added for cell lysis at 64 °C for 2 min. Lysates were cooled to 4 °C. RNA was extracted by the acid guanidinium thiocyanate-phenol-chloroform extraction method [28]. DNA contamination, if any, was eliminated using DNase I (NEB), which was deactivated by heating to 90 °C for 2 min.

### RNase T1 digestion

RNAs were trimmed with RNase T1 (Invitrogen) for 1 h.

### RNase H digestion

20-mer oligo-deoxy-ribonucleotides and buffer were added. The mixture was heated to 90 °C for 2min and cooled to room temperature naturally. RNase H (Thermo Scientific) was added for RNA digestion in DNA/RNA duplexes for 1 h. After 3 repeats, the oligonucleotides were removed by DNase I (NEB).

### RNA size selection

RNAs were resolved on 10% urea polyacrylamide gels. The bands corresponding to 40-100 nt were cut out and recovered using a ZR small-RNA PAGE recovery kit (Zymo research).

### RNA dephosphorylation

The recovered RNAs were incubated in a dephosphorylation mixture containing 8 U FastAP thermosensitive alkaline phosphatase (Thermo Scientific, EF0651) and 40 U RNase inhibitors in polynucleotide kinase (PNK) buffer for 45 min at 20 °C.

### RNA 5’ end phosphorylation and intramolecular ligation

RNAs were subsequently phosphorylated with 10 U of T4 polynucleotide kinase in PNK buffer (TAKARA) for 30 min at 37 °C. Cross-linked RNA molecules were then ligated using 40 U of T4 RNA ligase 1 (New England Biolabs, M0204), 1 mM ATP, and 40 U RNase inhibitors in RNA ligase 1 buffer for 1 h at 15 °C, and kept for 16 h at 4 °C.

### Photoreversal of cross-linking

For cross-linking reversal, the ligated RNAs were irradiated at 254 nm UV with a fluence of 400 mJ/cm^2^, followed by 200 mJ/cm^2^. RNAs were then precipitated overnight using isopropyl alcohol and washed twice with 75% alcohol.

### Library preparation and high-throughput sequencing

Sequencing libraries were generated using NEBNext® Ultra™ RNA Library Prep Kit for Illumina® (NEB, USA) following the manufacturer’s recommendations. Library preparation was carried out on an Illumina HiSeq 2000/2500 platform. To detect RRIs in *E. coli*, five samples were prepared. The sample with full treatment was termed ‘TAN’. Compared with the ‘TAN’ sample, ‘AN’ had no T4 RNA ligase treatment, while ‘A’ had no T4 RNA ligase and photoreversal treatments; ‘B’ was the sample without AMT, T4 RNA ligase and photoreversal treatments, while ‘U’ had no AMT, UV irradiation, T4 RNA ligase and photoreversal treatments.

### Bioinformatics analyses

The adapter sequences were removed from the raw sequencing reads using the Flexbar [29] software, which meanwhile could trim the 3’ end until the Phred quality score 30 or higher is reached. After that, those reads with length shorter than 10 nts, with undetermined bases taking up more than 10%, or with low quality score (<=5) bases taking up more than 50%, were filtered out. Then paired end reads were merged by a home-made program. In this study, the overlaps between paired end reads were no less than 10 nts. The reads were then mapped to the genome of *Escherichia coli* K12 MG1655 with BLAST [30]. Only BLAST hits without mismatches or gaps were considered. For each read, potential helical regions were predicted using GUUGle [31]. Then, chimeras (chimeric reads) were identified for subsequent analysis. Here we searched for “chimeras” satisfying the following criteria: (1) read not mapped continuously to the genome; (2) read generating two BLAST hits which together could cover it fully; (3) the two parts of the read (corresponding to the two BLAST hits) directly adjacent or having up to 4-nt overlap between them; (4) if the two BLAST hits were in the same gene, they should overlap each other in the gene; (5) the helical regions formed by the two parts of the read should contain at least one classical cross-linking site of AMT, i.e. 5’-UR or 5’-RU. The reads mapped to multiple gene pairs were discarded. Each combination of helical regions with AMT sites was used as constraint to assess the dimeric structure of the two parts by RNAcofold [4]. The structure with lowest energy was selected as the interaction structure frozen by AMT.

**Fig. 1:**
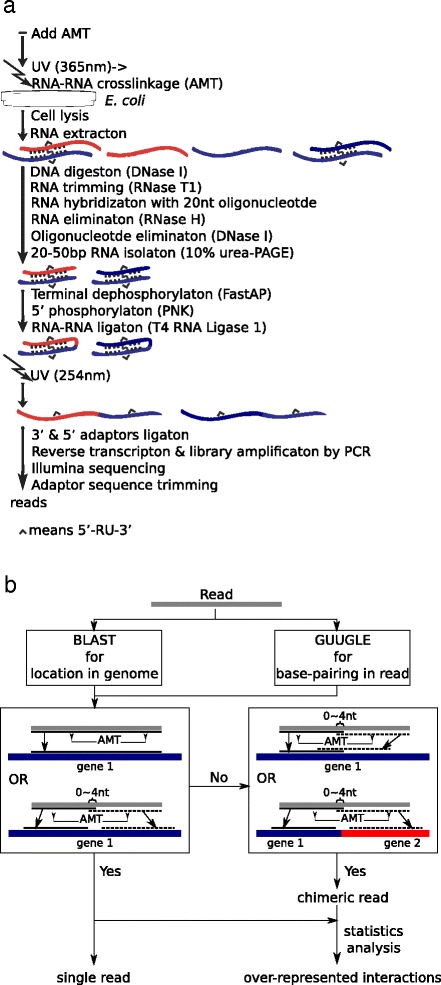
Schematic overview of the modified protocol. **a**, wet experiment. Irradiated with 365 nm UV, RNAs were cross-linked by AMT at the paired region, and survive DNase I, RNase T1 and RNase H treatments which digest DNA and single strand RNA. Cross-linked RNAs were ligated by T4 RNA ligase 1. After photoreversal of cross-linkages by 254 nm UV, the ligated RNAs could be sequenced and identified. **b**, bioinformatics analysis

Additionally, we defined the reads meeting the following criteria as “single reads”: (1) the read could be mapped continuously to the genome, and otherwise divided into two parts directly adjacent or with up to 4-nt overlap, mapped respectively to two nonoverlapping sequences within the same gene; (2) the helical regions should contain at least one classical cross-linking site of AMT, i.e. 5’-UR or 5’-RU. Reads mapped to multiple genes were discarded. Each combination of helical regions with AMT sites was used as constraint to derive the secondary structure of the read by RNAfold [4]. The folding with lowest energy was selected as the structure frozen by AMT. The reads mapped to multiple gene positions were discarded. The overall flowchart of the bioinformatics analysis was illustrated in Fig. 1b.

### Probabilistic analysis of inter-molecular interactions in chimeric reads

As the reads were composed of RNA sequences in different lengths corresponding to various genomic locations, the genome was divided evenly into non-overlapping windows, and these RNA sequences were named for the windows in which they appeared. Considering that different window sizes can affect the reads grouping accuracy and resolution, we tried several window sizes and checked the grouping results by drawing graphs. The window size of 100 demonstrated the most reasonable results. So we select 100 as the final window size for analysis. All single read- and chimeric partner- windows were counted as total RNAs, T_1_. Pairs of windows X and Y ligated by chimeric reads were counted. Window pairs (X, Y) that appeared in no less than ten chimeric reads were further analyzed. For a ligated window pair (X, Y), observed frequency was calculated as the number of pairs (X, Y) divided by the total number of chimeric reads. The expected frequency of random ligation was calculated as:

$$ 2\times \frac{N_x}{T_1}\times \frac{N_y}{T_1-1} $$

where N_x_ and N_y_ are counts of windows X and Y, respectively. In case of non-random ligation of windows X and Y, the observed frequency should be larger than the random frequency. Here, observed to expected frequency ratio for each window pair was calculated, and varied from 0.44 to 130.97. Then, window pairs with ratios > 2 were subjected to Fisher's exact test. Finally, chimeric reads of windows (X, Y) with *P* < 0.01 were identified as inter-molecular interactions frozen by AMT with statistical significance.

## Results

### Identification of ligated RNAs

This study (Fig. 1) employed in vivo crosslinking of RNA duplexes with the AMT molecule, which can, upon 365 nm UV irradiation, generate inter-strand adducts between juxtaposed uridine bases [32]. Following cell lysis and RNA extraction, DNA residues were digested by DNase I, and single strand RNAs and free RNA overhangs adjacent to duplexes were digested by RNase T1. Then, the residual single strand RNAs were hybridized with 20 nt oligonucleotides and digested by RNase H three times. Separated by urea PAGE electrophoresis, 40-100 nt RNAs were recovered for subsequent experiments. Free RNA overhangs adjacent to duplexes were ligated using T4 RNA ligase 1. After photoreversal of cross-linkages using 254 nm UV, the ligated RNAs were submitted to high-throughput sequencing and identified subsequently by bioinformatics methods (Fig. 1b). The sample was named ‘TAN’. To assess background, other four samples were prepared in parallel. Compared with the ‘TAN’ sample, ‘AN’ had no T4 RNA ligase treatment, while ‘A’ had no T4 RNA ligase and photoreversal treatments; ‘B’ was the sample without AMT, T4 RNA ligase and photoreversal treatments, while ‘U’ had no AMT, UV irradiation, T4 RNA ligase and photoreversal treatments. Finally, each sample yielded tens of millions of reads (Table 1).

**Table 1:** Statistics of reads from the five samples

Sample	Sequenced reads
U	12,163,535
B	12,398,653
A	16,023,462
AN	10,912,713
TAN	14,775,476

**Fig. 2:**
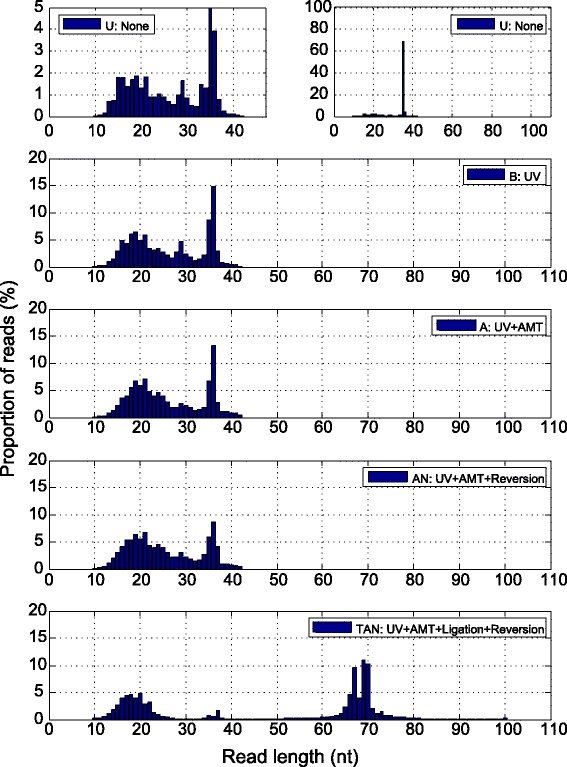
Length distribution of reads from various experiments, with different treatments. UV, 365 nm UV irradiation; AMT, AMT added; Ligation, treated with T4 RNA ligase; Reversion, photoreversal of cross-linking by 254 nm UV irradiation; None, none of the above treatments. For the ‘None’ group, the left sub-panel showed, for clarity, a 20x magnification view of a part of the right sub-panel

As expected, longer reads in the TAN sample emerged (Fig. 2). In samples U, B, A and AN, the reads were not longer than 42 nt, while in the TAN sample, 56.8% of the reads were longer than 42 nt, and should be originated from T4 RNA ligase treatment.

With mismatch and gap disallowed, each read from the TAN sample was mapped individually to the genome using the BLAST program [30]. Some reads mapped wholly to a continuous genomic sequence. They originated most likely from a single transcript, and thus were called single reads. Other reads could be split into two parts, with each one mapped wholly to a continuous genomic sequence. When the two genomic sequences corresponded to different transcripts, the term chimera was used for the read. When the two genomic sequences overlapped, the read was also called a chimera, which most likely originated from the interaction of two identical RNA molecules. When the two genomic sequences are separated in the same transcript, the read was defined as a single read. To eliminate chimeras originated from reverse transcription template switching, the two parts were required to be directly adjacent or with up to 4-nt gap or overlap in the read. Other reads should be split into more than two parts to ensure full mapping to the genome. These reads might originate from multiple ligations of RNA and/or cross-linked RNAs, which were not further analyzed in this study.

### The ligated RNAs form stable structures with low free energy distribution

**Fig. 3:**
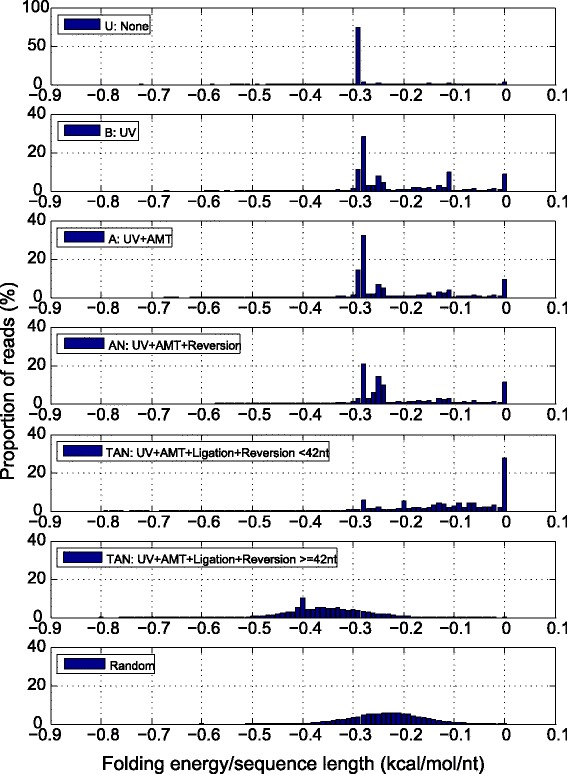
Distribution of normalized minimum folding energies of annotated reads from various samples. Different treatments are shown. UV, 365 nm UV irradiation; AMT, AMT added; Ligation, treated with T4 RNA ligase; Reversion, photoreversal of cross-linking by 254 nm UV irradiation; None, none of the above treatments; Random, random sequences from the genome. Minimum folding energies were calculated by RNAfold

If a read is composed of two RNA sequences which are cross-linked by AMT, the latter should form stable structures and thus possess low free energy. To test this hypothesis, free energy of secondary structures of reads from samples TAN, U, B, A, and AN were calculated using the RNAfold [4] program, and distribution is displayed in Fig. 3, in which free energies were normalized to the length of the corresponding read. The normalized folding energies of reads from samples U, B, A and AN were mostly between -0.3 ~ 0 kcal/mol/nt (Fig. 3). In the TAN sample, energies of reads shorter than 42 nt were also mostly between -0.3 ~ 0 kcal/mol/nt, whereas those of the remaining reads were obviously in the lower range of -0.48 ~ -0.2 kcal/mol/nt. Additionally, we generated a random sample of 10000 sequences of 40 ~ 100 nt in length; each of them was prepared by assembling a random number of fragments randomly selected from the *E. coli* genome, between the minimum and maximum number of ligated fragments in the sequenced TAN reads. Energies of the random sequences were mostly between -0.36 ~ -0.1 kcal/mol/nt. Finally, the difference of folding free energies between the 42 ~ 100 nt TAN sample and those from the random samples was calculated using *t*-test in R. A *P* value between the two samples was 2.2e-16, confirming that the long reads in the TAN sample were not originated from random ligation.

### Interactions in chimeric and single reads

We selected reads mapped uniquely for further analysis. As AMT was used to freeze the RNA interactions and enrich them for sequencing, the chimera should contain certain site pairs in both RNA sequences which could be recognized and linked by AMT. As for single reads, site pairs should also exist. We used GUUGle [31] and home-made programs to search classical cross-linking sites of AMT, i.e. 5’-UR or 5’-RU. Finally, 2652 chimeric and 11265 single reads were identified to contain reasonable cross-linking sites. For each read, there might be several possible combinations of base-paired regions containing AMT sites. We selected the one with lowest energy as constraint to determine the interaction’s structure of a chimera or non-continuous single read using RNAcofold, or the folding structure of a continuous single read using RNAfold.

### Identification of over-represented RNA-RNA interactions

To eliminate random ligated chimeras as much as possible, we performed statistical analysis to select RNA-RNA interactions significantly over-represented. For each RNA interaction pair supported by no less than 10 chimeras, observed and random expected frequencies were assessed. Pairs with observed to expected frequency ratios lower than 2 were filtered out. Then, Fisher’s exact test was applied to each pair count, and statistically significant ones (*p* < 0.01) were selected as putative interacting pairs. Finally, 32 interacting pairs from 29 pairs of transcripts were identified, which corresponded to 1082 chimeric reads (Table 2). Detailed information, such as original RNA positions, putative structures and interactions, are available at http://ccb1.bmi.ac.cn/htsrr/.

**Table 2:** Statistics of inter-molecular RNA-RNA interactions

Class pair	Reads	Gene pair
tRNA – tRNA	315	8 (glyT – glyU/proM/serV/thrT/tyrU, glyU – tyrU, tyrU-/valV/valW)
ncRNA – tRNA	266	7 (ffs – glyT/serV/tyrU, ssrS – glyT/glyU/hisR/proM)
tRNA – rRNA	199	5 (glyT – rrfA, proM – rrfA, serT – rrsG, serV – rrlA/rrlH)
rRNA – mRNA	141	2 (rrlC – cadA/rpoB)
rRNA – ncRNA	74	3 (ffs – rrfA/rrfF, ssrS – rrlH)
rRNA – rRNA	33	1 (rrsG – rrsG)
rRNA – IGT	28	1 (rrlC –)
tRNA – tmRNA	13	1 (serV – ssrA)
tRNA – IGT	13	1 (hisR –)
Total	1082	29

### Global snapshot of the *E. coli* RNA-RNA interactome

Among the detected single reads, 10697 reads were located in known genes, among which 10054 were located in 11 rRNA genes, 409 reads in 14 tRNA genes, 145 reads in 5 non-coding RNAs (ncRNAs), 73 reads in 58 mRNAs, 15 reads in transfer-messenger RNA (tmRNA) ssrA, and 1 read in pseudo mRNA yneO. For the remaining reads, 86 were located in intergenic regions, 472 crossed gene boundaries, 7 crossed 3 repeat region boundaries, and 3 were located in a repeat region named REP31. These reads may reflect new RNA transcripts, whose functional roles remain to be discovered.

The 1082 over-represented chimeric reads originated from different types of inter-molecular interactions (Table 2). For example, 315 reads were from 8 tRNA-tRNA interaction pairs, 266 from 7 ncRNA-tRNA pairs, 199 from 5 tRNA-rRNA pairs, 141 from 2 rRNA-mRNA pairs, 74 from 3 rRNA-ncRNA pairs, 33 from 1 rRNA-rRNA pair, and 13 from 1 tRNA-tmRNA pair. Interactions involving intergenic transcripts (IGTs) were also found, such as 28 reads from the interaction between rRNA rrlC and the transcript from a region between the protein coding genes udk and alkA, and 13 reads from that between tRNA hisR and the transcript from a downstream region of leuT. The existence of the novel transcript interacting with rrlC were proved by PCR and sequencing (Additional file 1: Document S1).

**Fig. 4:**
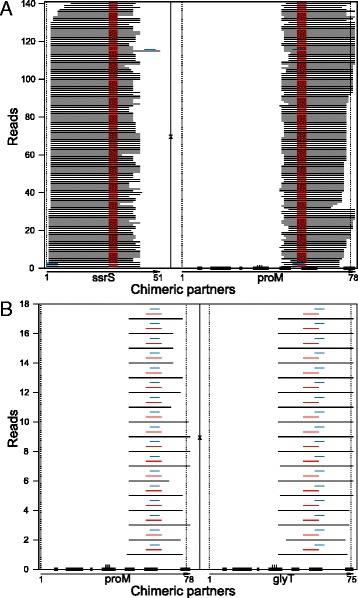
Possible base-pairing and cross-linking between the partners in chimeric reads. **a**, chimeric reads of ssrS and proM; **b**, chimeric reads of proM and glyT. The two chimeric partners are shown, respectively, in two parts of the panel, separated by a vertical line labeled “x”. Each sub-panel has its own horizontal coordinates to show the corresponding locations of partner RNA fragments in the parent gene transcript. For a parent transcript, unpaired sequences were marked by a thick line on the horizontal axis. Anticodons are shown as three short vertical lines. When the initial and/or end positions of a parent transcript are shown, they are also marked with vertical dot lines. For each read, the two partner RNA fragments are drawn horizontally as two black lines. Possible base-pairing and cross-linking sequences are marked above the lines with colored horizontal lines. Various colors were used to distinguish different possible combinations of the sequences

We totally found 29 interacting gene pairs (e.g. Fig. 4a). For each read corresponding to a certain interaction pair, all combinations of possible interacting sequences containing classical AMT cross-linking sites were drawn and compared. In the parent transcript, interaction sequences always appeared in or adjacent to a region not base-paired or wobble base-paired (Fig. 4), indicating that interaction partners tend to be in single-stranded regions.

Detailed information of the above reads can be accessed at our webserver http://ccb1.bmi.ac.cn/htsrr/. For each read, there is a webpage to illustrate its gene composition, base pairing position and sequence, interacting structure, and probability distribution of being unpaired in the parent genes; this will help related experimental research on RNA-RNA interactions and RNA structure.

## Discussion

In recent years, CLASH methods have been developed to detect RRIs in yeast [23], bacteria [24, 25] and human cells [26]. In these studies, UV was used to stimulate cross-linking between proteins and associated RNAs, and specific proteins were used to obtain the cross-linked RNAs for RRI detection. Although many RRIs have been discovered, they were limited to those associated with specific proteins, which did not cover the whole RNA-RNA interactome.

In this study, psoralen can intercalate into RNA duplexes and after irradiation with 320–400 nm light, uridines from the RNA duplex can be frozen by covalent attachment [33] when in close proximity [34]. When irradiated with 254 nm UV, crosslinking can be reversed. Psoralen induces intra- and inter-molecular cross-links within RNAs, it doesn’t matter if there exist other molecules such as proteins. This makes it possible to ligate all interacting RNA sequences, whether intra- or inter-molecular entities. Then, the concatenated RNA molecules can be used to prepare cDNA libraries for high-throughput sequencing, which should reveal the whole RNA-RNA interactome. To the best of our knowledge, this is the first study to scan the bacterial whole RNA-RNA interactome using a modified CLASH protocol. As expected, we detected both intra- and inter-molecular RNA-RNA interactions. Furthermore, crosslinking uridines preferred by AMT would be helpful to detect interacting sequences. To ensure reliability, the reads were analyzed and filtered strictly. For example, during mapping to the genome, no mismatch or gap was permitted; an interaction RNA pair should contain base-paired sequences with classical AMT cross-linking sites and be supported by no less than 10 reads. In addition, the count of an interaction RNA pair should be statistically significant. Although no sRNA-mRNA interaction was found with statistically significance in this study, almost all functional classes of RNAs were detected to be involved in various inter-molecular RNA-RNA interactions, among which tRNA-tRNA, tRNA-ncRNA, tRNA-rRNA, rRNA-mRNA, rRNA-ncRNA, rRNA-rRNA, rRNA-IGT and tRNA-IGT interactions were also detected in a RNase E-CLASH study by Waters et al [24]. 5 interacting gene pairs discovered in Waters’s study were detected with statistical significance in this study, of which 1 is tRNA-tmRNA and 4 is tRNA-tRNA. We detected not only alternative interaction regions in each of them, but also the same interaction regions in 3 tRNA-tRNA gene pairs. If we do not consider the statistical significance, additional 27 gene pairs revealed in Waters’s study were detected in this study, among which we detected the same interaction regions in 5, alternative interaction regions in 16, both same and alternative interaction regions in 6 (Additional file 2: List S1). The alternative interaction regions may demonstrate the dynamics of RNA-RNA interactions, which will affect and be affected by their interactions with other partner molecules. Detection of the same and alternative interaction regions showed the ability of this modified protocol to capture the dynamics of RNA-RNA interactions, no matter it occurred before, during or after the interaction with other partner molecules such as proteins. These results revealed an unexpected complexity of RNA-RNA interactions, even in a simple bacterial cell. These findings would benefit the functional researches of RNAs to explore the unexpected RNA-RNA interactions, especially their changes in various conditions. For example, among the identified 29 pairs of interacting genes, tRNAs appeared in 22, where tRNA, ncRNA, rRNA, tmRNA and an unknown transcript were partners. Here, tRNA fragments appearing as chimeras may be tRNA-derived fragments (tRFs) indeed. The tRFs have been described in all three domains of life, and are produced from mature tRNAs or their precursor transcripts; they serve as a source of small functional RNAs involved in many biological processes, such as translational inhibition and stress response. Research on tRFs is still in its beginning stage [35].

**Fig. 5:**
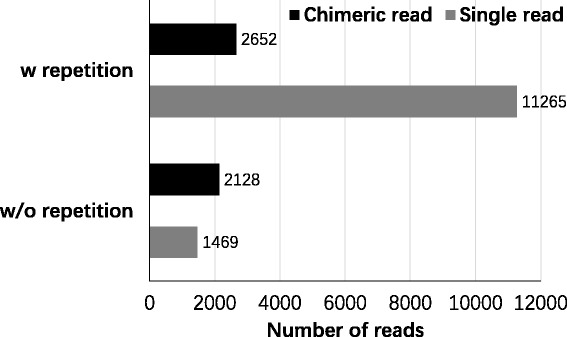
Statistics of uniquely mapped reads

As shown in Fig. 5, 1469 uniquely mapped single reads were obtained. These reads totally emerged as many as 11265 times. We also obtained 2128 inter-molecular chimeric reads. However, they totally emerged 2652 times, obviously lower than that in single reads. This may be consistent with constitutive intra-molecular interactions and transient regulatory inter-molecular interactions.

The known sRNA-mRNA interactions were not found, likely because: (1) sRNA-mRNA interactions are dependent on specific environmental changes. The experimental conditions employed in this study may not be consistent with those of known sRNA-mRNA interactions; (2) with the high abundance of rRNAs and tRNAs, the sequencing depth was not high enough, and known interactions of sRNA-mRNA could not be detected; (3) other reasons such as many experimental steps do not promote the detection of sRNA-mRNA interactions. In the next study, we will try to improve the protocol to detect low abundance of RNA-RNA interactions by simplifing the experimental processes, eliminating rRNAs and tRNAs, and increasing the sequencing depth. Moreover, we will apply it in the comparing studies of various conditions and strains to find the RNA-RNA interactions underlying various physiological and pathogenic phenomena.

Initially, we aimed to detect more sRNA-mRNA interactions using a modified CLASH protocol. Interestingly, another unexpected aspect of the complicated RNA-RNA interactions was revealed, indicating the necessity for studying RNA interactions broadly. The protocol applied here would be useful in future research for finding more RNA interactions and determining their interaction sequences. In addition, if combined with other CLASH protocols which can detect RRIs associated with proteins, we will be able to study the relation between the RNA-RNA interactome and the protein world.

## Conclusions

As demonstrated by the novel RNA-RNA interactions discovered in this study, there may still be multiple RNA-RNA interactions to be uncovered in *E. coli*. Methods for detecting various kinds of RNA-RNA interactions may be different. The present study provides a high-throughput protocol not only for discovering new RNA interactions, but also obtaining base-pairing sequences directly, which should be useful in assessing RNA structure and interactions.

## Additional files

Additional file 1: Document S1. PCR and sequencing results to demonstrate the existence of a novel transcript which was detected to interact with rRNA rrlC in this study. (PDF 1869 kb)

Additional file 2: List S1. List of interacting gene pairs detected in both this study and Waters’s study, including information of all reads mapped to these gene pairs. (TXT 28 kb)

## Abbreviations

AMT: 4’-aminomethyl-trioxsalen
CLASH: Cross-linking, ligation and sequencing hybrids
HITS: High-throughput sequencing
HITS-CLIP: High-throughput sequencing of RNAs isolated by crosslinking immunoprecipitation
IGT: Intergenic transcript
iPAR-CLIP: In vivo PAR-CLIP
ncRNA: Non-coding RNA
PAR-CLIP: Photoactivatable-ribonucleoside-enhanced crosslinking and immunoprecipitation
PNK: Polynucleotide kinase
RRI: RNA-RNA interaction
sRNA: Small regulatory RNA
tmRNA: Transfer-messenger RNA

## References

[1] Chou C-H, Chang N-W, Shrestha S, Hsu S-D, Lin Y-L, Lee W-H (2016). miRTarBase 2016: updates to the experimentally validated miRNA-target interactions database. Nucleic Acids Res.

[2] Lestrade L, Weber MJ (2006). snoRNA-LBME-db, a comprehensive database of human H/ACA and C/D box snoRNAs. Nucleic Acids Res.

[3] Zhang X, Wu D, Chen L, Li X, Yang J, Fan D (2014). RAID: a comprehensive resource for human RNA-associated (RNA-RNA/RNA-protein) interaction. RNA.

[4] Lorenz R, Bernhart SH, Höner ZU, Siederdissen C, Tafer H, Flamm C, Stadler PF (2011). ViennaRNA Package 2.0. Algorithms Mol Biol.

[5] Zuker M (2003). Mfold web server for nucleic acid folding and hybridization prediction. Nucleic Acids Res.

[6] Tafer H, Hofacker IL (2008). RNAplex: a fast tool for RNA-RNA interaction search. Bioinformatics.

[7] Thomas M, Lieberman J, Lal A (2010). Desperately seeking microRNA targets. Nat Struct Mol Biol.

[8] Li W, Ying X, Lu Q, Chen L (2012). Predicting sRNAs and their targets in bacteria. Genomics Proteomics Bioinformatics.

[9] Akhtar MM, Micolucci L, Islam MS, Olivieri F, Procopio AD (2016). Bioinformatic tools for microRNA dissection. Nucleic Acids Res.

[10] Liu B, Li J, Cairns MJ (2014). Identifying miRNAs, targets and functions. Brief Bioinform.

[11] Agarwal V, Bell GW, Nam JW, Bartel DP (2015). Predicting effective microRNA target sites in mammalian mRNAs. Elife.

[12] Krek A, Grün D, Poy MN, Wolf R, Rosenberg L, Epstein EJ (2005). Combinatorial microRNA target predictions. Nat Genet.

[13] Kertesz M, Iovino N, Unnerstall U, Gaul U, Segal E (2007). The role of site accessibility in microRNA target recognition. Nat Genet.

[14] Miranda KC, Huynh T, Tay Y, Ang Y-S, Tam W-L, Thomson AM (2006). A pattern-based method for the identification of MicroRNA binding sites and their corresponding heteroduplexes. Cell.

[15] Rehmsmeier M, Steffen P, Hochsmann M, Giegerich R (2004). Fast and effective prediction of microRNA/target duplexes. RNA.

[16] Wright PR, Georg J, Mann M, Sorescu DA, Richter AS, Lott S (2014). CopraRNA and IntaRNA: predicting small RNA targets, networks and interaction domains. Nucleic Acids Res.

[17] Eggenhofer F, Tafer H, Stadler PF, Hofacker IL (2011). RNApredator: fast accessibility-based prediction of sRNA targets. Nucleic Acids Res.

[18] Kery MB, Feldman M, Livny J, Tjaden B (2014). TargetRNA2: identifying targets of small regulatory RNAs in bacteria. Nucleic Acids Res.

[19] Cao Y, Zhao Y, Cha L, Ying X, Wang L, Shao N (2009). sRNATarget: a web server for prediction of bacterial sRNA targets. Bioinformation.

[20] Ying X, Cao Y, Wu J, Liu Q, Cha L, Li W (2011). Starpicker: A method for efficient prediction of bacterial sRNA targets based on a Two-Step model for hybridization. PLoS ONE.

[21] Chi SW, Zang JB, Mele A, Darnell RB (2009). Argonaute HITS-CLIP decodes microRNA-mRNA interaction maps. Nature.

[22] Hafner M, Landthaler M, Burger L, Khorshid M, Hausser J, Berninger P (2010). Transcriptome-wide identification of RNA-binding protein and microRNA target sites by PAR-CLIP. Cell.

[23] Kudla G, Granneman S, Hahn D, Beggs JD, Tollervey D (2011). Cross-linking, ligation, and sequencing of hybrids reveals RNA-RNA interactions in yeast. Proc Natl Acad Sci U S A.

[24] Waters SA, McAteer SP, Kudla G, Pang I, Deshpande NP, Amos TG (2017). Small RNA interactome of pathogenic E. coli revealed through crosslinking of RNase E. EMBO J.

[25] Melamed S, Peer A, Faigenbaum-Romm R, Gatt YE, Reiss N, Bar A (2016). Global Mapping of Small RNA-Target Interactions in Bacteria. Mol Cell.

[26] Helwak A, Kudla G, Dudnakova T, Tollervey D (2013). Mapping the human miRNA interactome by CLASH reveals frequent noncanonical binding. Cell.

[27] Grosswendt S, Filipchyk A, Manzano M, Klironomos F, Schilling M, Herzog M (2014). Unambiguous identification of miRNA:target site interactions by different types of ligation reactions. Mol Cell.

[28] Chomczynski P, Sacchi N (1987). Single-step method of RNA isolation by acid guanidinium thiocyanate-phenol-chloroform extraction. Anal Biochem.

[29] Dodt M, Roehr JT, Ahmed R, Dieterich C (2012). FLEXBAR-Flexible Barcode and Adapter Processing for Next-Generation Sequencing Platforms. Biology (Basel).

[30] Altschul SF, Gish W, Miller W, Myers EW, Lipman DJ (1990). Basic local alignment search tool. J Mol Biol.

[31] Gerlach W, Giegerich R (2006). GUUGle: a utility for fast exact matching under RNA complementary rules including G-U base pairing. Bioinformatics.

[32] Calvet JP, Pederson T (1979). Heterogeneous Nuclear-Rna Double-Stranded Regions Probed in Living Hela-Cells By Crosslinking With the Psoralen Derivative Aminomethyltrioxsalen. Proc Natl Acad Sci U S A.

[33] Wassarman DA (1993). Psoralen crosslinking of small RNAs in vitro. Mol Biol Rep.

[34] Cantor CR, Wollenzien PL, Hearst JE (1980). Structure and topology of 16S ribosomal RNA. An analysis of the pattern of psoralen crosslinking. Nucleic Acids Res.

[35] Shigematsu M, Honda S, Kirino Y (2014). Transfer RNA as a source of small functional RNA. J Mol Biol Mol imaging.

