# Supplementary material for: Detecting RNA-RNA interactions in E. coli using a modified CLASH method
Source: BMC Genomics. 2017 May 3;18:343. doi: 10.1186/s12864-017-3725-3 (PMC5415748; doi:10.1186/s12864-017-3725-3)
Supplement: Supplementary file 1 — Document S1. PCR and sequencing results to demonstrate the existence of a novel transcript which was detected to interact with rRNA rrlC in this study. (PDF 1869 kb) [file 12864_2017_3725_MOESM1_ESM.pdf]

1. Reads of rrlC and an intergenic transcript. Genome coordinates are based on the genome of *E. coli* K12 MG1655 (NC_000913.3). In read id, the last number means the count of the read.

Read ID	RNA1	Strand	Genome coordinates		RNA2	Strand	Genome coordinates	
			From	To			From	To
TAN_5386294_2	rrlC	+	3943989	3944052	IGT	-	2143278	2143291
TAN_6571173_2	rrlC	+	3943991	3944052	IGT	-	2143278	2143291
TAN_2642600_2	rrlC	+	3943992	3944052	IGT	-	2143278	2143291
TAN_7788407_2	rrlC	+	3943993	3944052	IGT	-	2143278	2143291
TAN_4128736_2	rrlC	+	3943994	3944052	IGT	-	2143278	2143291
TAN_157415_1	rrlC	+	3943995	3944052	IGT	-	2143279	2143291
TAN_1767244_5	rrlC	+	3943995	3944052	IGT	-	2143278	2143291
TAN_10182757_1	rrlC	+	3943996	3944052	IGT	-	2143279	2143291
TAN_1331712_8	rrlC	+	3943996	3944052	IGT	-	2143278	2143291
TAN_988601_3	rrlC	+	3943997	3944052	IGT	-	2143278	2143291

2. Primers.

Primer pair 1
Forward primer GTCCAGGTCATGTTCAGCGA Genome coordinates:2143546 2143527
Reverse primer GGATGTTAGGAAGGGGGCG Genome coordinates:2142972 2142990
Product length 575

Primer pair 2
Forward primer GGCGATAAAACGCCACCATC Genome coordinates:2143443 2143424
Reverse primer CCAAACGCGGCAATTATAGGG Genome coordinates:2142999 2143019
Product length 445

3. Gel electrophoresis of PCR products. P1 and P2 are the products of primer pair 1 and 2 respectively.

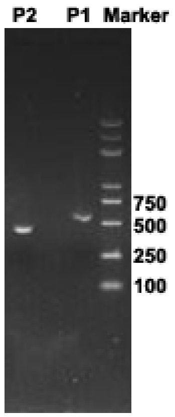

4. Sequences of PCR products and their Blast results against the genome.

Product of primer pair 1:

TTTTCGTACTGATGGATGCGGCGATATTTCCCAGCGAACAGCTCAGCGCAATTCCCGGCCACATGCGCCCGGCATGGCGA
TAAAACGCCACCATCATGATGGACGTCGGGAACCACAGTGGGGCGAGTTGGGTGCCAAACTGCGAAAGCTCAAGCGAGAA
AAGGGTAAAGATAAACGAGACTAAACCTAAACTGACCAGGTGAAGCAGCGGGTGGGGCAGGGCAATTAATACATGCTGTG
ATTGTTTGCTCATTACCGCTTTATCCGCAAGACCTGGTCGCCAGTATTCGTTTCTGTGTTTATGCTAGTACAAAAGTTTT
ACATTTTATATGCGAATTGCTCATAAAGTGACGTAAAGGCGGATAATTTGCGCAACTGCGTTTAACATTTTTTACCTTAC
ATAAAACTGATCAACGTAATTTGCCCCCGGGAATTCTTTGTCAACAATTAACTCTGAGCGCGCGGCAACGCTATTCGACT
GGTATCAGACGGATGAAATCCCTATAATTGCCGCGTTTGGCGCTTCGTCGCCCCCTTCCTAACATCCA

Chromatogram:

File: BJ17030301296(5)5-F(zidai)_Py_E12.ab1 Run Ended: Mar 4, 2017, 5:34:23 Signal G:499 A:509 T:396 C:398
Sample: BJ17030301296(5)5-F(zidai)_Py Lane: 88 Base spacing: 16.72 548 bases in 6627 scans
Page 1 of 1

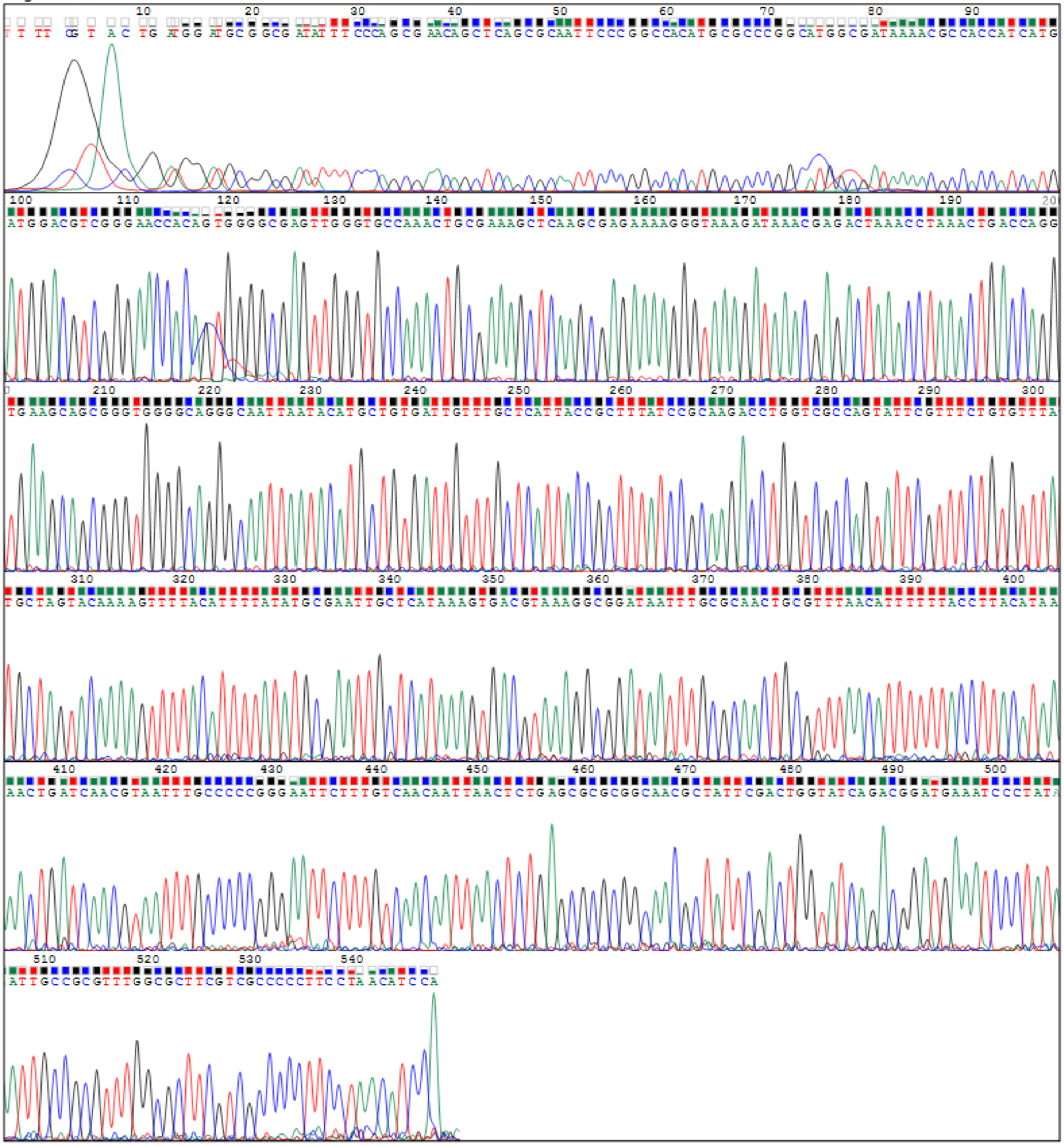

Blast result:

Download GenBank Graphics

Escherichia coli str. K-12 substr. MG1655, complete genome

Sequence ID: NC_000913.3 Length: 4641652 Number of Matches: 1

Range 1: 2142971 to 2143508 GenBank Graphics Next Match Previous Match

Score	Expect	Identities	Gaps	Strand
994 bits(538)	0.0	538/538(100%)	0/538(0%)	Plus/Minus

```
Query  11       GATGGATGCGGCGATATTTCCCAGCGAACAGCTCAGCGCAATTCCCGGCCACATGCGCCC  70
                ||||||||||||||||||||||||||||||||||||||||||||||||||||||||||||
Sbjct  2143508  GATGGATGCGGCGATATTTCCCAGCGAACAGCTCAGCGCAATTCCCGGCCACATGCGCCC  2143449

Query  71       GGCATGGCGATAAAACGCCACCATCATGATGGACGTCGGGAACCACAGTGGGGCGAGTTG  130
                ||||||||||||||||||||||||||||||||||||||||||||||||||||||||||||
Sbjct  2143448  GGCATGGCGATAAAACGCCACCATCATGATGGACGTCGGGAACCACAGTGGGGCGAGTTG  2143389

Query  131      GGTGCCAAACTGCGAAAGCTCAAGCGAGAAAAGGGTAAAGATAAACGAGACTAAACCTAA  190
                ||||||||||||||||||||||||||||||||||||||||||||||||||||||||||||
Sbjct  2143388  GGTGCCAAACTGCGAAAGCTCAAGCGAGAAAAGGGTAAAGATAAACGAGACTAAACCTAA  2143329

Query  191      ACTGACCAGGTGAAGCAGCGGGTGGGGCAGGGCAATTAATACATGCTGTGATTGTTTGCT  250
                ||||||||||||||||||||||||||||||||||||||||||||||||||||||||||||
Sbjct  2143328  ACTGACCAGGTGAAGCAGCGGGTGGGGCAGGGCAATTAATACATGCTGTGATTGTTTGCT  2143269

Query  251      CATTACCGCTTTATCCGCAAGACCTGGTCGCCAGTATTCGTTTCTGTGTTTATGCTAGTA  310
                ||||||||||||||||||||||||||||||||||||||||||||||||||||||||||||
Sbjct  2143268  CATTACCGCTTTATCCGCAAGACCTGGTCGCCAGTATTCGTTTCTGTGTTTATGCTAGTA  2143209

Query  311      CAAAAGTTTTACATTTTATATGCGAATTGCTCATAAAGTGACGTAAAGGCGGATAATTTG  370
                ||||||||||||||||||||||||||||||||||||||||||||||||||||||||||||
Sbjct  2143208  CAAAAGTTTTACATTTTATATGCGAATTGCTCATAAAGTGACGTAAAGGCGGATAATTTG  2143149

Query  371      CGCAACTGCGTTTAACATTTTTTACCTTACATAAAACTGATCAACGTAATTTGCCCCCGG  430
                ||||||||||||||||||||||||||||||||||||||||||||||||||||||||||||
Sbjct  2143148  CGCAACTGCGTTTAACATTTTTTACCTTACATAAAACTGATCAACGTAATTTGCCCCCGG  2143089

Query  431      GAATTCTTTGTCAACAATTAACTCTGAGCGCGCGGCAACGCTATTCGACTGGTATCAGAC  490
                ||||||||||||||||||||||||||||||||||||||||||||||||||||||||||||
Sbjct  2143088  GAATTCTTTGTCAACAATTAACTCTGAGCGCGCGGCAACGCTATTCGACTGGTATCAGAC  2143029

Query  491      GGATGAAATCCCTATAATTGCCGCGTTTGGCGCTTCGTCGCCCCCTTCCTAACATCCA  548
                ||||||||||||||||||||||||||||||||||||||||||||||||||||||||||
Sbjct  2143028  GGATGAAATCCCTATAATTGCCGCGTTTGGCGCTTCGTCGCCCCCTTCCTAACATCCA  2142971
```

Product of primer pair 2:

GCCGAACATCATACCAGTGGGGCGAGTTGGGTGCCAAACTGCGAAAGCTCAAGCGAGAAAAGGGTAAAGATAAACGAGAC
TAAACCTAAACTGACCAGGTGAAGCAGCGGGTGGGGCAGGGCAATTAATACATGCTGTGATTGTTTGCTCATTACCGCTT
TATCCGCAAGACCTGGTCGCCAGTATTCGTTTCTGTGTTTATGCTAGTACAAAAGTTTTACATTTTATATGCGAATTGCT
CATAAAGTGACGTAAAGGCGGATAATTTGCGCAACTGCGTTTAACATTTTTTACCTTACATAAAACTGATCAACGTAATT
TGCCCCCGGGAATTCTTTGTCAACAATTAACTCTGAGCGCGCGGCAACGCTATTCGACTGGTATCAGACGGATGAAATCC
CTATAATTGCCGCGTTTGGA

Chromatogram:

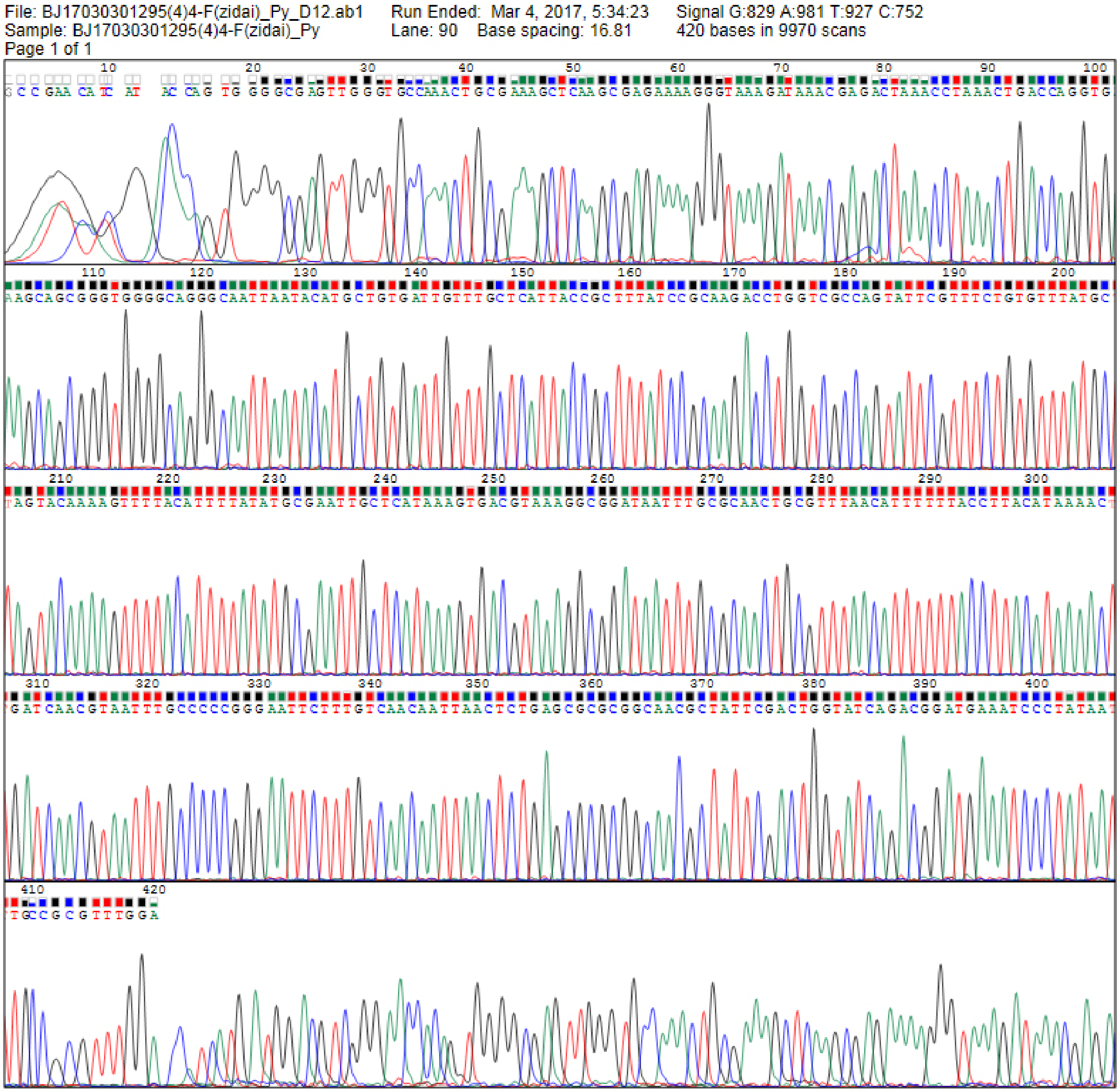

Blast result:

Download GenBank Graphics

Escherichia coli str. K-12 substr. MG1655, complete genome

Sequence ID: NC_000913.3 **Length:** 4641652 **Number of Matches:** 1

Range 1: 2142999 to 2143403 GenBank Graphics

Next Match Previous Match

Score	Expect	Identities	Gaps	Strand
749 bits(405)	0.0	405/405(100%)	0/405(0%)	Plus/Minus

```
Query  15       CAGTGGGGCGAGTTGGGTGCCAAACTGCGAAAGCTCAAGCGAGAAAAGGGTAAAGATAAA  74
                ||||||||||||||||||||||||||||||||||||||||||||||||||||||||||||
Sbjct  2143403  CAGTGGGGCGAGTTGGGTGCCAAACTGCGAAAGCTCAAGCGAGAAAAGGGTAAAGATAAA  2143344

Query  75       CGAGACTAAACCTAAACTGACCAGGTGAAGCAGCGGGTGGGGCAGGGCAATTAATACATG  134
                ||||||||||||||||||||||||||||||||||||||||||||||||||||||||||||
Sbjct  2143343  CGAGACTAAACCTAAACTGACCAGGTGAAGCAGCGGGTGGGGCAGGGCAATTAATACATG  2143284

Query  135      CTGTGATTGTTTGCTCATTACCGCTTTATCCGCAAGACCTGGTCGCCAGTATTCGTTTCT  194
                ||||||||||||||||||||||||||||||||||||||||||||||||||||||||||||
Sbjct  2143283  CTGTGATTGTTTGCTCATTACCGCTTTATCCGCAAGACCTGGTCGCCAGTATTCGTTTCT  2143224

Query  195      GTGTTTATGCTAGTACAAAAGTTTTACATTTTATATGCGAATTGCTCATAAAGTGACGTA  254
                ||||||||||||||||||||||||||||||||||||||||||||||||||||||||||||
Sbjct  2143223  GTGTTTATGCTAGTACAAAAGTTTTACATTTTATATGCGAATTGCTCATAAAGTGACGTA  2143164

Query  255      AAGGCGGATAATTTGCGCAACTGCGTTTAACATTTTTTACCTTACATAAAACTGATCAAC  314
                ||||||||||||||||||||||||||||||||||||||||||||||||||||||||||||
Sbjct  2143163  AAGGCGGATAATTTGCGCAACTGCGTTTAACATTTTTTACCTTACATAAAACTGATCAAC  2143104

Query  315      GTAATTTGCCCCCGGGAATTCTTTGTCAACAATTAACTCTGAGCGCGCGGCAACGCTATT  374
                ||||||||||||||||||||||||||||||||||||||||||||||||||||||||||||
Sbjct  2143103  GTAATTTGCCCCCGGGAATTCTTTGTCAACAATTAACTCTGAGCGCGCGGCAACGCTATT  2143044

Query  375      CGACTGGTATCAGACGGATGAAATCCCTATAATTGCCGCGTTTGG  419
                |||||||||||||||||||||||||||||||||||||||||||||
Sbjct  2143043  CGACTGGTATCAGACGGATGAAATCCCTATAATTGCCGCGTTTGG  2142999
```
